# AP-2δ Expression Kinetics in Multimodal Networks in the Developing Chicken Midbrain

**DOI:** 10.3389/fncir.2021.756184

**Published:** 2021-10-21

**Authors:** Lutz Kettler, Hicham Sid, Carina Schaub, Katharina Lischka, Romina Klinger, Markus Moser, Benjamin Schusser, Harald Luksch

**Affiliations:** ^1^Chair of Zoology, Technical University of Munich, Freising, Germany; ^2^Reproductive Biotechnology, Technical University of Munich, Freising, Germany; ^3^Institute for Biology I, Faculty of Biology, University of Freiburg, Freiburg, Germany; ^4^TranslaTUM, Technical University of Munich, Munich, Germany

**Keywords:** AP-2, chicken, inferior colliculus, optic tectum, shepherd’s crook neuron, multimodal, brain development

## Abstract

AP-2 is a family of transcription factors involved in many aspects of development, cell differentiation, and regulation of cell growth and death. AP-2δ is a member of this group and specific gene expression patterns are required in the adult mouse brain for the development of parts of the inferior colliculus (IC), as well as the cortex, dorsal thalamus, and superior colliculus. The midbrain is one of the central areas in the brain where multimodal integration, i.e., integration of information from different senses, occurs. Previous data showed that AP-2δ-deficient mice are viable but due to increased apoptosis at the end of embryogenesis, lack part of the posterior midbrain. Despite the absence of the IC in AP-2δ-deficient mice, these animals retain at least some higher auditory functions. Neuronal responses to tones in the neocortex suggest an alternative auditory pathway that bypasses the IC. While sufficient data are available in mammals, little is known about AP-2δ in chickens, an avian model for the localization of sounds and the development of auditory circuits in the brain. Here, we identified and localized AP-2δ expression in the chicken midbrain during embryogenesis. Our data confirmed the presence of AP-2δ in the inferior colliculus and optic tectum (TeO), specifically in shepherd’s crook neurons, which are an essential component of the midbrain isthmic network and involved in multimodal integration. AP-2δ expression in the chicken midbrain may be related to the integration of both auditory and visual afferents in these neurons. In the future, these insights may allow for a more detailed study of circuitry and computational rules of auditory and multimodal networks.

## Introduction

The vertebrate midbrain is a central hub for fast visual and multimodal orientation in complex environments (Basso and May, 2017; Herman et al., 2018). In addition to the superior colliculus of mammals, the optic tectum (TeO) of birds has been studied in great detail (Luksch, 2003; Wylie et al., 2009) due to several advantages: a distinctly laminated architecture with 15 layers (Cajal, 1911), a relatively large size and a well-described embryogenesis (Thanos and Mey, 2001). Furthermore, the separation of input and output layers facilitates analysis: visual input reaches the superficial layers 2–7 (Yamagata et al., 2006), whereas output originates from the deeper layers 9–15 (Reiner and Karten, 1982; Hellmann and Güntürkün, 2001). The visual input is retinotopic and transfers a visual map of space onto the tectal surface (Hunt and Webster, 1975; Yuasa et al., 1996), with different classes of retinal ganglion cells innervating specific retinorecipient layers (Sanes and Yamagata, 1999; Yamagata et al., 2006) where they synapse onto specific tectal cell types that are developmentally dependent on this input (Lischka et al., 2018b).

The TeO does not only process visual information, but also receives auditory input from the midbrain *nucleus mesencephalicus lateralis pars dorsalis* (Mld), which is mostly termed inferior colliculus (IC) to align with the mammalian counterpart (Knudsen, 1982). Whereas the visual tectal map results from a simple coordinate transfer from the retina, the localization of an acoustic signal needs to be computed from binaural cues along the auditory pathway: interaural time and intensity difference (ITD and IID; Konishi, 2003; Wagner et al., 2013). The processing of ITDs and IIDs along the auditory pathway have mostly been analyzed in the barn owl, where they are processed separately starting at the level of the first auditory nucleus, i.e., *nucleus magnocellularis* and *nucleus angularis*, and converge in the lateral shell of the IC (Konishi, 2003). Finally, in the external nucleus of the IC (ICx), auditory spatial receptive fields are formed as an auditory map of space (Knudsen and Konishi, 1978). This map is then projected onto the TeO *via* direct and indirect connections (Peña and Gutfreund, 2014; Niederleitner et al., 2017). Bimodal cells in the TeO have both a small visual receptive field and a much larger auditory spatial receptive field (Knudsen, 1982). This integration is dynamic and can be altered by sensory manipulations, with the visual system being dominant (reviewed in Knudsen and Brainard, 1995). Multimodal integration facilitates and accelerates the detection of weak stimuli in behavior (Whitchurch and Takahashi, 2006; Verhaal and Luksch, 2016).

In the precocial chicken, all developmental steps must lead to a functional network before hatching. The chicken is a suitable and well-studied model for the cellular and molecular processes that establish the connections in the retino-tectal projection (Thanos and Mey, 2001; Kukreja et al., 2017) and the early auditory pathway (Kubke and & Carr, 2000). AP-2 is an important family of transcription factors implicated in many aspects of development, cell differentiation, and the regulation of cell growth and death (Hilger-Eversheim et al., 2000). The AP-2 transcription factors are sequence-specific DNA binding proteins that have been identified in various species including Drosophila, Xenopus, chicken, mouse, and human (Shen et al., 1997; Monge and Mitchell, 1998; Hilger-Eversheim et al., 2000; Luo et al., 2005). Modulation of the concentration and activity of these proteins provides a fundamental mechanism for regulating gene expression (Zhao et al., 2001). All members of the AP-2 family bind with varying affinity to GC-rich elements, and contain a proline- and glutamine-rich domain in the N-terminal half which mediates transcriptional activation (Williams et al., 1988; Wankhade et al., 2000; Werling and Schorle, 2002). Five AP-2 genes (*Tcfap2*) have been isolated in mice and humans: AP-2α, AP-2β, AP-2γ, AP-2δ (*Tcfap2d*), and AP-2ε (Moser et al., 1995; Zhao et al., 2001). AP-2δ is highly similar to other AP-2 proteins in the DNA-binding and dimerization domains, however, there is significant divergence in the N-terminal transactivation domain, where AP-2δ lacks residues that are critical for transcriptional activation in the other members of this family (Zhao et al., 2001; Eckert et al., 2005). Among the eight residues in the transactivation domain deemed critical for AP-2 function, only three are conserved in AP-2δ (Li et al., 2008). AP-2δ is predominantly expressed in the midbrain and at lower levels in the diencephalon, forebrain, spinal cord, and the retina and for a short period in the developing heart (Zhao et al., 2003). AP-2δ is an important transcription factor, specifying gene expression patterns required for the development of the posterior midbrain in mice (Hesse et al., 2011). Li et al. (2008) showed that in the chicken embryo, AP-2δ is primarily expressed in the retina and brain with the highest levels at embryonic days 7–11, and that AP-2δ RNA and protein are found in a subset of ganglion cells in the embryonic chick retina.

In the brain of the adult mouse, AP-2δ is expressed in the posterior midbrain (inferior colliculus), as well as in the cortex, dorsal thalamus, and the mammalian counterpart to the TeO, the superior colliculus (SC; Hesse et al., 2011). AP-2δ deficient mice are viable but lack part of the inferior colliculus due to increased apoptosis in this part of the brain, starting at the end of embryogenesis (Hesse et al., 2011). Despite the absence of the inferior colliculus, AP-2δ deficient mice appear to retain at least some higher auditory function, as neural responses to sounds can still be recorded in the auditory cortex, likely through auditory information bypassing the inferior colliculus (Hesse et al., 2011; Schofield et al., 2014). However, hodological studies of the AP-2δ deficient mice have not been performed so far.

Here, we investigated the expression profile of AP-2δ in chicken during development. As the neuronal circuitry in the chicken midbrain is well described, lesioning of the inferior colliculus and subsequent analysis of the anatomical, physiological, and behavioral effects might provide valuable clues on the integration of auditory input in the midbrain. We were also interested in identifying possible AP-2δ—positive elements in the TeO, as many cell types of the chicken TeO are characterized, and the labeling of a specific cell type might yield further insight into the developmental role of AP-2δ.

## Material and Methods

### Animals

Fertilized eggs of White Leghorn chicken (*Gallus gallus domesticus*) were obtained from the in-house chicken facility TUM School of Life Sciences, Weihenstephan, Germany and incubated in a breeder (38.2°C temperature, 50% humidity) until the desired embryonic developmental stage (Hamburger and Hamilton, 1951). Chickens were fed with a commercial diet *ad libitum* and had free access to water all the time. All animal work was in accordance with the German Animal Protection Act.

### RT-PCR

AP-2δ expression was investigated in different organs including the midbrain, forebrain, brainstem, heart, liver, breast muscle, and intestine. The RNA from different chicken organs at different developmental stages (E10, E14, E18) and post hatch day 2 (P2) was isolated by using the Reliaprep™ RNA Tissue Miniprep System according to the manufacturer’s instructions (Promega, USA) and controlled for purity and concentration. cDNA synthesis was conducted using the GoScript Reverse transcription mix (Promega, USA).

AP-2δ expression was detected with primers 900_ AP-2δ_fw2 (5’-CGTCCACGATGCAGAGATACG-3’) and 901_ AP-2δ_rev2 (5’-CGGTGCCCGTGGTAGAATAAG-3’) resulting in a 137 bp amplicon. ß-actin was detected with primers β-actin_F (5′-TACCACAATGTACCCTGGC-3′) and β-actin_R (5′-CTCGTCTTGTTTTATGCGC-3′) resulting in a 300 bp amplicon. The PCR reaction was performed using FIREPol DNA Polymerase (Solis Biodyne, Estonia) according to manufacturer’s instructions and done with the following thermal profile: 95°C for 5 min, followed by 35 cycles of 95°C for 30 s 59°C for 30 s 72°C for 20 s and a final elongation step at 72°C for 5 min.

### Western Blot

For Western Blot analysis, the midbrains of various embryonic stages were isolated, snap-frozen in liquid nitrogen, and stored at −80°C until further use. The tissue was lysed with RIPA Buffer added to the tissue samples and homogenized. Samples were incubated for 2 h at 4°C on a rotator and centrifuged for 20 min at 4°C and 12,000 rpm. The supernatant was transferred to a fresh tube and stored at −80°C. Protein concentration was estimated by the bicinchoninic acid (BCA) assay. The BSA calibration line was prepared, starting from a BSA stock solution between 200 and 1,000 μg/ml BSA. BCA Working Reagent and the samples were transferred into a 96-well plate. After 30 min of incubation at 37°C, the BSA dilutions were measured spectrophotometrically at 562 nm and then the unknown protein samples were determined by standard series.

Protein separation was achieved with a 10% SDS-polyacrylamide gel electrophoresis (SDS-PAGE). To achieve a good separation performance of the electrophoresis, extracted proteins were focused on a stacking gel before separation. For sample preparation, 15 μg of protein and the appropriate volume of 6× Laemmli-buffer supplemented with β-Mercaptoethanol were added to a final concentration of 1× sample buffer and subsequently heated for 5 min at 95°C and 300 rpm. After incubation, the samples were shortly centrifuged and applied to the completely polymerized gel. The visualization of the protein size was done by applying 6 μl of PageRuler™ Plus Prestained Protein Ladder (Thermo Fisher Scientific, USA). The gel electrophoresis was done using SDS buffer at a constant voltage of 80 mV for stacking gel or 200 mV for separating gel.

Blotting onto nitrocellulose membranes was carried out at a constant voltage of 80 V for 45 min. The membranes were then incubated with a blocking solution (3% BSA) on a shaker for 1 h at room temperature. The incubation of the anti-AP-2δ antibody (and separately with anti-ß-actin as housekeeping control) was carried out overnight at 4°C in TBS-T. Subsequently, membranes were washed with TBS-T buffer (3 × 10 min) and incubated with the secondary antibody (Alexa Fluor™ 546 goat anti rabbit IgG, 2 mg/ml) diluted (1:5,000) in TBS-T for 1 h at room temperature in dark. The membranes were then washed six times for 5 min with TBS-T at room temperature and inspected with a fluorescence microscope (see below).

### Immunohistochemistry

Embryos (E6–E20) were taken from the egg and euthanized. The brain was extracted and immediately put into ice-cold paraformaldehyde solution [4% in 0.1 M phosphate buffer (PB)]. After overnight fixation, brains were cryoprotected and sectioned in the transversal plane to 60 μm on a freezing microtome (Microme HM 400 E, GMI, USA). Sections were collected and rinsed three times in PBS (0.1 M PB with 0.75% NaCl) before incubation for 10 min in 0.5% H_2_O_2_ in 75% Methanol to block endogenous peroxidases. After several washing steps in PBS, an antigen retrieval step was performed that consisted of incubation in 10 mM sodium citrate buffer (pH 8.5) for 20 min at 60°C in a water bath. After cooling to room temperature, sections were again rinsed three times in PBS. The tissue was then incubated in a blocking solution (1 h at room temperature) containing 5% normal goat serum (NGS, Linaris S-1000, Cat# 0163.2) and 0.5% Triton X-100 (Tx100, Fluka) to avoid cross-binding. Afterward, the tissue sections were incubated with the polyclonal anti-AP-2δ that was used in mice (Hesse et al., 2011, diluted 1:2,500 in PB) overnight at 4°C. After several washing steps, antibody binding was visualized by incubation with an avidin-biotin peroxidase complex (ABC; Vectastain Elite ABC Kit, Vector Laboratories Inc., Burlingame, CA, USA; 3.2 ml/ml) in PBS-Tx100 (0.5%/4% NaCl) for 2 h. After washing in PB and acetate imidazole buffer (AIP, 0.175 M acetate, 0.069% imidazole, pH 7.4/6.5), tissue was pre-incubated in a 0.025% diaminobenzidine solution (DAB-buffer tablets for microscopy, Merck KGaA, Darmstadt, Germany) with 1% NiSO_4_ in AIP (pH 6.5) for 5 min and the chromogenic reaction was induced by adding H_2_O_2_ (end concentration 0.0025%) for 3 min. After washing the tissue, the sections were mounted on gelatin-coated slides, counterstained with neutral red, and coverslipped with DPX (DPX Mountant for Histology, Sigma-Aldrich GmbH, Steinheim, Germany).

### Double Labeling in Slices

To obtain double labeled structures with both AP2-δ-IH and traced connections within the tectal network, we performed slice experiments in six embryos (E14, E16, E18). Animals were taken from the egg and decapitated. After decapitation, the brain was extracted, and the midbrain was isolated and separated along the midline. The hemispheres were embedded in agarose (low-melting-point agarose, Sigma, USA, 2% in HEPES buffer Sigma, USA) and horizontally sliced at 500–1,000 μm with a vibratome (VF-100, Precisionary Instruments, Greenville, NC). The slices were collected in a chamber filled with ACSF solution (120 mM NaCl, 3 mM KCl, 1 mM MgCl_2_, 23 mM NaHCO_3_, 1.2 mM NaH_2_PO_4_, 2 mM CaCl_2_, 11 mM D-glucose; pH 7.4) and continuously oxygenated with carbogen at room temperature. Small crystals of biotinylated dextran amine (BDA, MW 3000, 10% in PB, Invitrogen,) were applied with a fine needle into the *nucleus isthmi parvocellularis* (Ipc, three embryos) or the *nucleus geniculatus lateralis pars ventralis* (Glv, three embryos) under visual control. After application, slices were incubated in oxygenated ACSF for 4 h to allow transport of the tracer. Slices were fixed in 4% PFA for 2 h and subsequently transferred to 30% sucrose (w/v in 0.1 M PB) overnight for cryoprotection before resectioning to 40 μm on a microtome (Microm HM440E, GMI, USA). Labeled structures were visualized with a streptavidin coupled to Alexa 546 (1:500 in 0.1 M PBS with 0.5% Tx100, Molecular Probes, USA). The sections were then treated to the immunohistochemistry protocol as above, and visualized with an Alexa Fluor 488 goat anti-rabbit, 1:500, Molecular Probes, USA) directed against the primary rabbit AP-2δ antibody. After 2 h of incubation at room temperature, sections were washed and mounted under a coverslip with n-Propylgallat (0.2%, diluted in DMSO, Glycerol, and PB).

### Microscopic Analysis

All sections were scanned with a fluorescence microscope equipped with a color camera (DP26, Olympus, Japan) for bright field microscopy of DAB-developed sections and a grayscale camera (XM10, Olympus, Japan) was used for fluorescent Western blot membranes. The microscope and both cameras were controlled with the CellSense Dimension software (Olympus, Japan).

To analyze the colocalization of AP-2δ on neurons prelabeled with retrograde tracing, we took fluorescence images stacks with a confocal laser scanning microscope (Leica TCS SP8, Leica Microsystems, Germany) using a 10× or a 40× objective. Image stacks were imported to FIJI (ImageJ 1.52i). Here, fluorescence channels were separated, filtered with a mean 3D filter [2 2 2], and adjusted for contrast and brightness. The stacks were reduced and focused using the “stack focuser” plugin (ImageJ, open-source plugin by Mikhail Umorin). Afterward, channels were merged to obtain an overlaid image of the retrogradely labeled neurons and AP-2δ in a maximum projection. Cells were counted using the *Cell detection* tool from QuPath (QuPath, Open-source Quantitative Pathology and Bioimage Analysis v.0.30, Bankhead et al., 2017) with default cell detection parameters for fluorescence images. Cell counts and double labeling between E14 and E18 were statistically analyzed using the Matlab (Mathworks, Natick, MA) statistics toolbox. For ANOVA analysis degrees of freedom (df) and F-statistic were calculated followed by the Tukey-Kramer *post hoc* test.

## Results

### Time Course of AP-2δ Expression During Development

**Figure 1:**
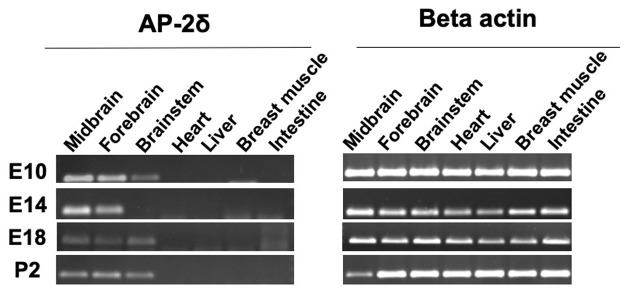
RT-PCR analysis of AP-2δ. RNA was isolated by using Reliaprep™ Tissue Miniprep System. **(Left panel)** AP-2δ expression in various tissues is demonstrated by a 137-bp PCR amplicon. Data shown for embryonic day 10–18 (E10–E18) and post-hatch day 2 (P2). **(Right panel)** β-actin (300 bp, left) serves as a control.

We were able to detect the AP-2δ expression by RT-PCR at different embryonic stages as well as after hatching in the midbrain, forebrain, and brainstem (Figure 1). Overall, no expression was detected in organs beyond the brain including the heart, liver, breast muscle, and intestine. AP-2δ expression was strong in the midbrain and forebrain at E10 and E14, decreasing in the later stages (E 18 and post hatch day 2, P2). The brainstem had a rather low level of expression throughout these stages, with a gap at E14.

**Figure 2:**
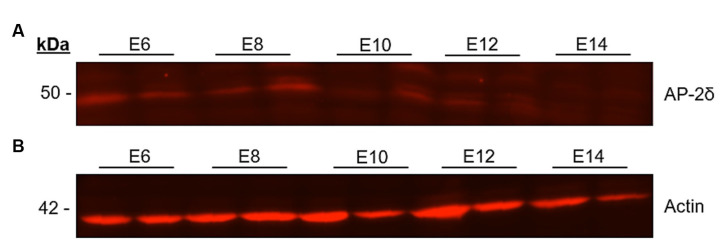
Western blot analysis of AP-2δ. Midbrains of E6–E14 chicken embryos were lysed with RIPA buffer and proteins were separated in a 10% SDS-PAGE gel and transferred to a nitrocellulose membrane. The gel was loaded with lysate. **(A)** To detect the AP-2δ (50 kDa), a rabbit anti-AP-2δ antibody and an Alexa Fluor™ 546 goat anti rabbit IgG (H + L) secondary antibody were used. **(B)** Loading control with rabbit anti-ε-Actin antibody (42 kDa).

On the protein level, the Western blot showed a band at 50 kDa for AP-2δ at early stages E6 to E14. The intensity was higher at the early stages and decreased after E10 (Figure 2A). In addition to the band at 50 kDa, several other extraneous bands were very lightly stained (data not shown), and at 42 kDa a single band was detected for the β-actin loading control (Figure 2B).

### AP-2δ Is Exclusive to the Auditory Midbrain and Tectal Layer 10

**Figure 3:**
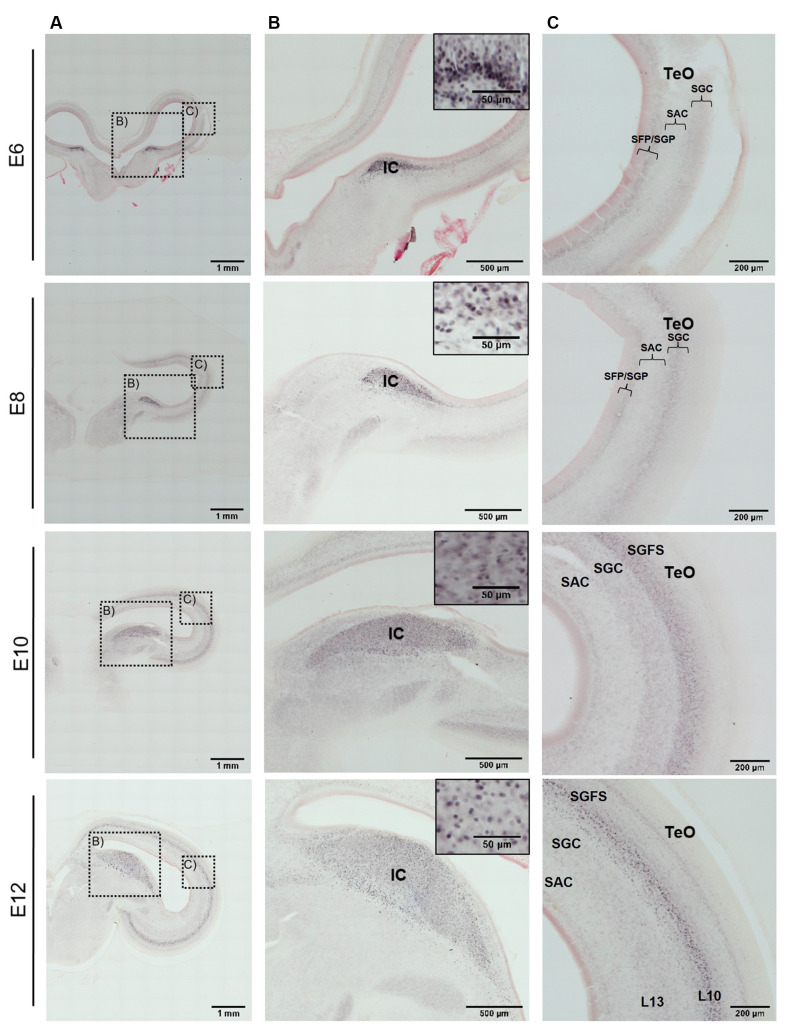
Immunohistochemistry against AP-2δ in chicken midbrains of E6–12 embryos. Frontal slices with DAB staining and neutral red counterstaining. **(A)** Overview of midbrain hemisphere, scale bar 1 mm. **(B)** Labeling in the inferior colliculus (IC) and cell nuclei staining (inset). Scale bars 500 μm and 50 μm (inset). **(C)** Labeling in the optic tectum (TeO). SFP, *stratum fibrosum periventriculare*; SGP, *stratum griseum periventriculare*; SAC, *stratum album centrale*; SGC, *stratum griseum centrale*; SGFS, *stratum griseum et fibrosum superficiale*. Scale bar 200 μm.

In the developing chicken midbrain, immunohistochemistry against AP-2δ revealed a clear label in various structures of the auditory inferior colliculus and the optic tectum (Figure 3A). At the earliest stage investigated (E6), the prospective inferior colliculus (IC, Figure 3B) was already visible as an elongated, thin zone of strongly immunopositive cells at the transition between the optic tectum and the tegmentum. In the tectum, laminae were not differentiated at that stage, but the neuroepithelium showed a diffuse label (Figure 3C).

Two days later at E8, the IC was clearly defined, with a sharp boundary towards the medial aspects. In the optic tectum, cell differentiation had begun and an intermediate layer of cell bodies (termed layer II according to Lavail and Cowan, 1971) was weakly immunopositive for AP-2δ in the upper aspects (Figure 3 E8). This situation became more pronounced at E10, where the IC had strongly increased in size and remained intensely labeled. Layering in the TeO had developed to a point where the prospective *stratum griseum et fibrosum superficiale* (SGFS) could be differentiated and could clearly be identified as the immunopositive layer in the TeO (Figure 3 E10).

**Figure 4:**
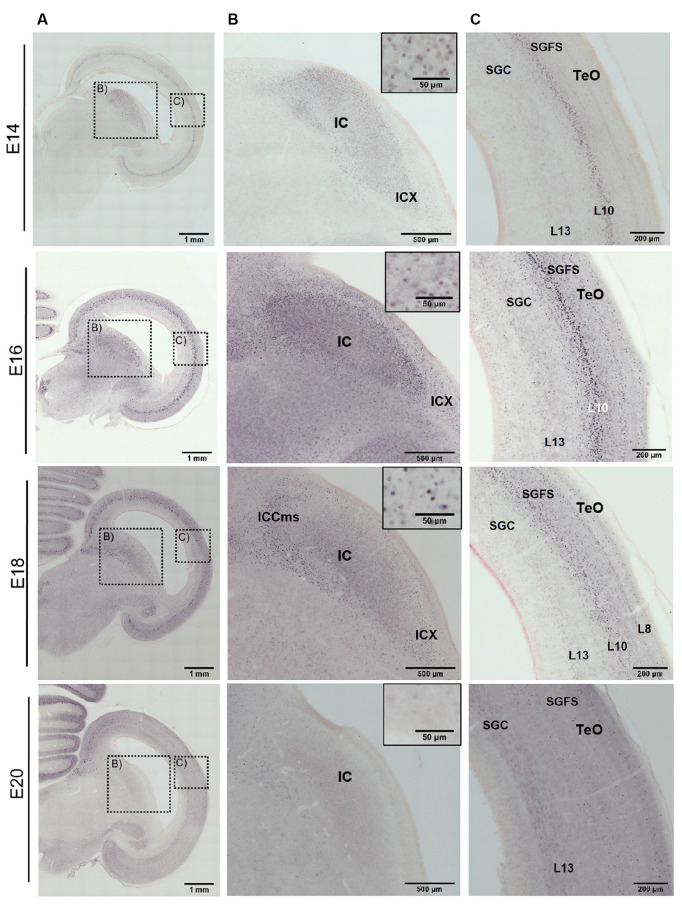
Immunohistochemistry against AP-2δ in chicken midbrains of E14–20 embryos. DAB staining, counterstain neutral red. **(A)** Frontal section of midbrain hemisphere. Scale bar 1 mm. **(B)** Labeling in the inferior colliculus (IC) and cell nuclei staining (inset). ICX, external nucleus of the IC; ICCms, medial shell of the central nucleus of the IC. Scale bars 500 μm and 50 μm. **(C)** Labeling in the optic tectum (TeO). SGC, *stratum griseum centrale*, SGFS, *stratum griseum et fibrosum superficiale*. Scale bar 200 μm.

At E12 and E14, the labeling pattern did not change much. The IC became increasingly larger and formed subzones that were not further analyzed (Niederleitner and Luksch, 2012). In the TeO, labeling was clearly located in layer 10 of the SGFS (Figure 3 E12, Figure 4 E14). Labeling of AP-2δ at E16 and E18 continued to be strong in the IC. However, the more medial aspects of the IC (medial shell of the IC and *area intercollicularis*) became intensely labeled (Figures 4A,B E16 and E18). Also, at E16, labeling in the core zone of the IC started to decrease, while the region described as the external nucleus of the IC (ICx, Niederleitner and Luksch, 2012, Figure 4B E16) retained the labeling seen in earlier embryonic stages. At E16 and E18, layer 10 of the TeO remained strongly labeled, but additional dispersed cells occurred in the upper layers (layers 4 and 8; Figure 4C E16 and E18). Finally, at E20, labeling intensity against AP-2δ had strongly decreased throughout the midbrain. Only a few neurons in the medial aspects towards the IC remained immunopositive, and in the TeO few dispersed neurons in layers 10, 8, and 4 retained their label (Figure 4C E20).

**Figure 5:**
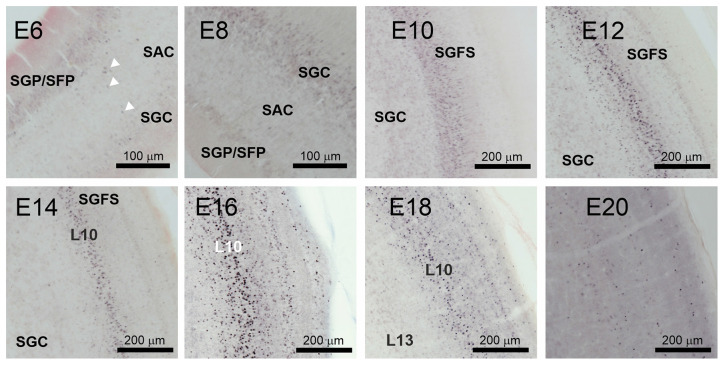
AP-2δ expression during optic tectum layer formation. DAB labeling of AP-2δ immunopositive cells from E6 to E20 in the developing optic tectum. Panels (E6–E8) show periventricular layers (SGP/SFP), marginal fiber layer (*SAC, stratum album centrale*), and granular layer (*SGC, startum griseum centrale*). White arrowheads mark AP-2δ positive migratory neuroblasts in the E6 SAC. Scale bar 100 μm. Panels (E10–20) illustrate formation and stratification of the *stratum griseum et fibrosum superficiale* (SGFS). Scale bars 200 μm.

A closer look at the tectal neuropil (Figure 5) revealed that in E6 AP-2δ immunopositive neuroblasts were found radially migrating from the *stratae griseae et fibrosae periventriculares* (SGP/SFP) through the *stratum album centrale* (SAC) to the *stratum griseum centrale* (SGC) consistent with early stratification of the TeO (Watanabe and Yaginuma, 2015). In E8 when the *stratum griseum et fibrosum superficiale* (SGFS) is still not defined most immunopositive neurons concentrate in the SGC. As apparent in the tectal overviews (Figure 3C) SGFS starts forming at E10 where now most of the AP-2δ positive cells were found. However, in the subsequent development, the majority of the labeled cells aggregate in layer 10 while the neurons in layer 13 of the SGC did not express AP-2δ. In E12 granular layer 8 and in E14 granular layer 4 begin to form (Watanabe and Yaginuma, 2015) and also contain labeled cells until expression almost ceases in any layer at E20. The aggregation of AP-2δ expression in the IC and the visual pathway (Li et al., 2016) indicated an influence of the transcription factor in auditory pathway and multimodal circuit formation. Hence, we tested which layer 10 cell types are expressing AP-2δ.

### Tectal Shepherd’s Crook Neurons Express AP-2δ

**Figure 6:**
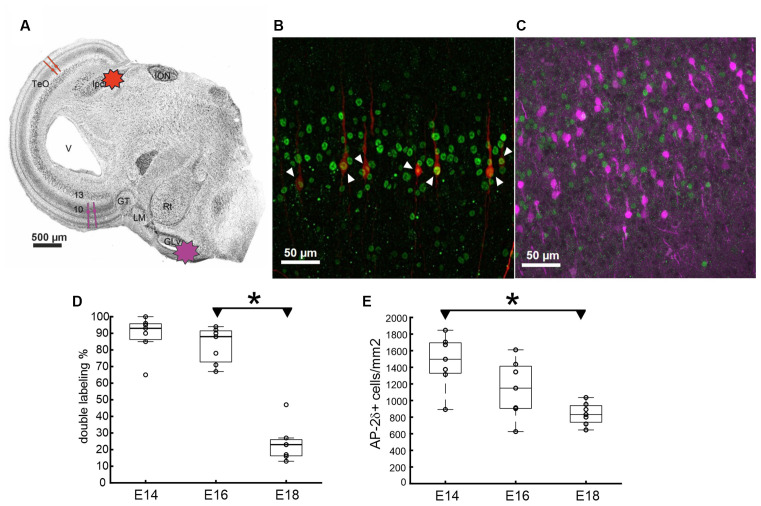
Localization of AP-2δ expression in the optic tectum (TeO). **(A)** Chicken brain slice showing the sectioning angle and the schematic injection areas for the tracer to label different cell populations in layer 10 of the TeO. **(B,C)** Double labeling with BDA tracing from either the Ipc (**B**, red) or the Glv (**C**, violet) in combination with AP-2δ labeling (green) in an E16 brain slice. Co-localization of BDA tracer and AP-2δ in shepherd’s crook neurons (SCN) indicated by double labeling in **(B)** (white arrows). No co-localization was found after BDA injection into Glv. ION, *Nucleus isthmo-opticus*; Ipc, *N. isthmi parvocellularis*; Glv, *N. geniculatus lateralis pars ventralis*; GT, *Griseum tectalis*; LM, *N. lentiformis mesencephali*; Rt, *N. rotundus*. **(D)** Percentage of double labeled SCN neurons at E14–E18 with individual data points at *n* = 7 random slice sections from one brain at each embryonic state. Zero double labeled cells in layer 10 (L10) after BDA injection in Glv. **(E)** AP-2δ immunopositive cell density for the same sections (area 0.975 mm^2^, depth 40 μm) as in **(D)**. Boxplots show median and interquartile range (asterisks: Tukey-Kramer multiple comparison test, *p* < 0.01).

Double labeling to further identify the cells in layer 10 of the TeO that express AP-2δ was performed in slice preparations with varying orientations (Figure 6). Figure 6A exemplifies the BDA injection sites in an oblique section. Note that the tracing for SCNs, however, was performed in transverse sections and the figure panel is for illustration purposes only. Shepherd’s crook neurons in layer 10 were readily retrogradely labeled at E16 (Lischka et al., 2018b) by BDA injection into the Ipc. Double labeling with immunocytochemistry against AP-2δ clearly showed that every labeled shepherd’s crook neuron was also positive for AP-2δ (Figure 6B). In contrast, labeling of tectal neurons projecting to the thalamic *nucleus geniculatus lateralis pars ventralis* (Glv) was performed in oblique sections as described in (Vega-Zuniga et al., 2014; Figure 6A). These retrogradely labeled “vine” neurons (Vega-Zuniga et al., 2014) did not show immunoreactivity for AP-2δ, indicating that layer 10 vine neurons do not express this transcription factor at E16 (Figure 6C). We further analyzed the number of co-localizations in E14, E16, and E18 SCN neurons (Figure 6D) and found a significant decrease in double labeling from E16 to E18 (ANOVA, *df* = 2, *F* = 71.72, *p* < 0.001 and Tukey-Kramer multiple comparison test, *p* < 0.001). Counting the double labeled cells in seven randomized sections of 0.975 mm^2^ and 40 μm thickness per embryonic stage across the TeO revealed that almost all cells (median 93%) that were identified as SCN by BDA labeling were also AP-2δ immunopositive at E14 and still 88% were double labeled at E16. The percentage dropped between E16 and E18 with only 23% SCN left being AP-2δ labeled. The overall density of AP-2δ positive cells (Figure 6E) in 40 μm thick brain slices significantly (ANOVA, *df* = 2, *F* = 8.76, *p* < 0.01 and Tukey-Kramer multiple comparison test, *p* < 0.01) decreased from E14 (1,497 cells/mm^2^) to E16 (831 cells/mm^2^). These data are consistent with the observations from the DAB-stained sections, where the density of AP-2δ labeled cells clearly decreased from E16 to E20 (Figure 4C).

## Discussion

The midbrain is a subcortical area involved in important functions such as multimodal integration, movement initiation, bottom-up attention, and stimulus selection. In precocial animals like the chicken, the crucial circuitry must be established prior to hatching. We analyzed the expression of the transcription factor AP-2δ in the chicken, which has previously been shown to be expressed strongly in the developing auditory midbrain of mice (Hesse et al., 2011). Our data show strong AP-2δ expression in the chicken auditory midbrain from the early stages of development and expression decreases towards hatching. Interestingly, we also detected AP-2δ expression in the optic tectum in a cell population that is involved in multimodal integration.

### AP-2δ Expression During Chicken Development

Among all investigated organs, AP-2δ was exclusively expressed in the Midbrain, forebrain, and brainstem. This provides new information regarding AP-2δ expression in the central nervous system of a phylogenetically distant species from mammals (Hesse et al., 2011). In order to determine the AP-2δ expression at the embryonal stages E6–E14 in the chicken midbrain, Western blot analysis was performed using a polyclonal rabbit anti-AP-2δ antibody. The polyclonal rabbit anti-AP-2δ antibody was produced in rabbits (Hesse et al., 2011) by coupling an AP-2δ specific peptide (GSQYGMHPDQRLLPG) to Maleimide Activated mcKLH (Pierce), and used in mice (Hesse et al., 2011; Li et al., 2016) This amino acid sequence is identical in both humans and chicken AP-2δ (Li et al., 2008), and the antibody should thus bind to the epitope in the chicken and human. The protein detected at 50 kDa corresponds to the molecular weight of AP-2δ and the band mainly found in other studies (Li et al., 2008) was detected at every embryonic stage analyzed. However, a few weaker side bands were also labeled (Figure 2A). To investigate the localization of the AP-2δ expression in the chicken brain at different stages, immunohistochemical staining with a polyclonal rabbit anti-AP-2δ antibody was performed and yielded clear labeling in the cell nuclei (Eckert et al., 2005) at every stage investigated. However, labeling intensity was stronger between E6 and E18, with a clear decline at embryonic day E20 prior to hatching. This transient expression profile during development corresponds to the transient expression of AP-2δ observed in the chicken retina where expression peaks around E10 and is already decreased at E15 (Li et al., 2008).

Within the midbrain of the developing chick, the expression of AP-2δ clearly defined the inferior colliculus starting at E6. Even at that early time point, the IC forms a slight bulge in the developing tegmentum and is linked up with the neuroepithelial zone of the TeO, which has not yet generated any layers at that time. The IC continues to express AP-2δ almost during the entire embryogenesis, enlarging and, at E12, beginning to show subzones that can be differentiated through subtle differences in the AP-2δ label. While the differentiation of the IC subdivision was not the focus of this analysis, it should be noted that there is an ongoing discussion on the subdivisions of the IC in birds, a discussion that might profit from a thorough analysis of AP-2δ expression (Puelles et al., 1994; Wang and Karten, 2010; Niederleitner and Luksch, 2012). This is even more relevant as, starting at E16, parts of the intercollicular zone medial to the IC (Wang et al., 2017) were also expressing AP-2δ. At embryonic day 18, the labeling intensity in the IC decreased and the AP-2δ signal at E20 was almost absent, indicating that the expression window of AP-2δ has closed. In addition to the AP-2δ expression in the IC, specific staining of cell nuclei is observed in the TeO. At E6, layering of the TeO has not started, and staining was seen in the neuroepithelial layer, which retains some signal at E8 where the first separate lamina (lamina II according to Lavail and Cowan, 1971) has formed and is also lightly stained. At later stages, this label can clearly be attributed to layer 10 of the SGFS, which is formed by E10 (Watanabe and Yaginuma, 2015). The AP-2δ expression continued to get stronger until embryonic day 16, was reduced at E18, and was almost absent at E20, except for some dispersed cell nuclei in layers 10, 8, and 4.

### Identification of Tectal Neurons

Layer 10 of the chicken TeO consists of several radial cell types, two of which have been analyzed in respect to their connectivity: shepherd’s crook neurons (SCN) and vine neurons (VN). SCNs receive visual input from retinal ganglion cells and auditory input from the IC *via* the *formatio reticularis lateralis* (FRLx; Niederleitner et al., 2017; Lischka et al., 2018a). SCN axons project to the isthmic nuclei (Garrido-Charad et al., 2018). VNs receive input from retinal ganglion cells and form an axon, which projects topographically to the GLv (*nucleus geniculatus lateralis pars ventralis*, Vega-Zuniga et al., 2014). To determine which of these cell types expresses AP-2δ during development, we performed double label experiments that clearly identified the SCN as the labeled population. The onset of the expression coincides well with the migration of SCN precursor cells, which is finished at embryonic day 10 when the gross morphology of these cells has been established (Domesick and Morest, 1977).

### AP-2δ Expression During Development in Vertebrates

AP-2 proteins are higher-order transcription factors that control a variety of downstream transcription factors, suppressing apoptosis, and controlling cell differentiation in various tissues (Hilger-Eversheim et al., 2000; Eckert et al., 2005). In our study, we investigated the expression and distribution of AP-2δ in chicken development and found a clear transient expression profile in the inferior colliculus, which was strongest between E6 and E16. Interestingly, a specific tectal projection neuron likely involved in multimodal integration also expresses AP-2δ in the same time window.

A comparable developmental pattern of AP-2δ expression is also observed in mammals. Hesse et al. (2011) showed with *in situ* hybridization in mice that at E12.5 and E14.5 AP-2δ is localized to the mesencephalic superior colliculus and the dorsal diencephalon. A more intense AP-2δ signal became apparent in the posterior midbrain (precursor to the inferior colliculus) at E14.5. Even more striking was this shift in AP-2δ expression towards the posterior midbrain at later stages of development that reached its apex around hatching. In the adult mouse brain, AP-2δ was predominantly expressed in the inferior colliculus. Additionally, weak AP-2δ expression was detectable in the dorsal thalamus and the forebrain both during embryogenesis and adulthood (Hesse et al., 2011). Moreover, Hesse et al. (2011) disrupted the *Tcfap2d* gene in mice and the results showed that AP-2δ deficient mice are viable but lack part of the posterior midbrain due to increased apoptosis in this part of the brain starting at the end of embryogenesis. Mice deficient in AP-2δ appear to retain at least some higher auditory function, as neuronal responses to sounds were recorded in the neocortex, suggesting an alternate auditory route that allows response to individual tones, despite the absence of the inferior colliculus (Hesse et al., 2011). Schofield et al. (2014) later confirmed projections from auditory hindbrain and subcollicular midbrain to thalamic auditory relays in the medial geniculate nucleus.

While the role of AP-2δ in the inferior colliculus is likely to suppress apoptosis, it is unclear why this transcription factor is specifically expressed in some tissues and not in others. The expression of AP-2δ in both the auditory midbrain and cochlear hair cells (Luo et al., 2019) might suggest a role in auditory network formation, but it is also expressed in the visual system, specifically in the retina (Li et al., 2008) where approximately one-third of ganglion cells are AP-2δ-positive. Loss of AP-2δ leads to reduced ganglion cell number and altered retinal projection to the superior colliculus in mice (Li et al., 2016), and ectopic expression of AP-2δ in the chicken results in misrouting of the axons, apparently by altering the polysialylation of NCAM (Li et al., 2014). Interestingly, we found in our study that a specific cell type of the chicken optic tectum, the SCN, also expresses AP-2δ. SCN receives both auditory and visual inputs and are among the earliest bimodal cells found in the chicken brain, feeding into a network that constitutes a bottom-up system for spatial location determination and attentional allocation. Whatever the functional reason might be, a correlation between AP-2δ expression and early sensory processing appears to exist.

### Outlook

What is the functional role of AP-2δ expression in the sensory processing networks of the midbrain? To answer this, manipulation of the expression may give important cues. In a series of experiments, Hesse et al. (2011) generated AP-2δ deficient mice by disruption of the *Tcfap2d* gene. These mice were viable but lacked part of the posterior midbrain due to increased apoptosis in the inferior colliculus of the brain starting at the end of embryogenesis. Despite the absence of the IC, a central part of the auditory pathway, AP-2δ deficient mice retained auditory function in the neocortex. This suggests an alternate auditory route that possibly allows responses to individual tones (Hesse et al., 2011; Schofield et al., 2014).

As both in chicken and mice AP-2δ is expressed in homologous regions during development and disruption of the *Tcfap2*d in mice led to an ablation of the IC, future efforts could be directed towards generating AP-2δ knockout chicken germlines that possibly develop selective lesions of the auditory midbrain. The layout of the chicken midbrain is very well known, and the visual and auditory processing networks have been delineated in great detail (Luksch, 2003; Wylie et al., 2009; Wang et al., 2017; Lischka et al., 2018a). This could open up tremendous opportunities for network analysis and understanding of multimodal integration in the midbrain.

## Data Availability Statement

The original contributions presented in the study are included in the article, further inquiries can be directed to the corresponding author.

## Ethics Statement

Ethical review and approval was not required for the animal study because the killing of animals solely for the purpose of organ removal or cell harvesting is not considered an animal experiment and only requires a declaration but no approval by an animal ethics committee according to the German Animal Welfare Act.

## Author Contributions

All authors had full access to all the data in the study and take responsibility for the integrity of the data and the accuracy of the data analysis. Study concept and design: HL and BS. Acquisition of data: LK, CS, KL, RK, HS, and HL. Analysis and interpretation of data: LK, CS, HL, HS, and BS. Drafting of the manuscript: LK, CS, HS, and HL. Critical revision of the manuscript for important intellectual content: LK, HL, HS, MM, and BS. Study supervision: HL and BS. All authors contributed to the article and approved the submitted version.

## Conflict of Interest

The authors declare that the research was conducted in the absence of any commercial or financial relationships that could be construed as a potential conflict of interest.

## Publisher’s Note

All claims expressed in this article are solely those of the authors and do not necessarily represent those of their affiliated organizations, or those of the publisher, the editors and the reviewers. Any product that may be evaluated in this article, or claim that may be made by its manufacturer, is not guaranteed or endorsed by the publisher.
